# Pathways for Cardioprotection in Perspective: Focus on Remote Conditioning and Extracellular Vesicles

**DOI:** 10.3390/biology12020308

**Published:** 2023-02-14

**Authors:** Stefano Comità, Chiara Rubeo, Magalì Giordano, Claudia Penna, Pasquale Pagliaro

**Affiliations:** Department of Clinical and Biological Sciences, University of Turin, Regione Gonzole 10, Orbassano, 10043 Torino, TO, Italy

**Keywords:** cardioprotective strategies, ischemia/reperfusion injury, postconditioning, preconditioning, remote conditioning

## Abstract

**Simple Summary:**

Despite the development of cutting-edge treatments, coronary artery disease (CAD) morbidity and mortality rates remain present at high levels. New cardioprotective approaches are crucial to improve the health of patients. Remote ischemic conditioning (RIC) seems to be the most promising method for heart repair. Recently, it has been shown that small, anuclear, bilayered lipid membrane particles, known as extracellular vesicles (EVs), are the drivers of signal transduction in either cardiac ischemia/reperfusion injury (IRI) or RIC. We will discuss how EVs can be used as a new drug delivery mechanism and how they can be employed in cardiac treatment.

**Abstract:**

Despite the development of cutting-edge treatments, coronary artery disease (CAD) morbidity and mortality rates remain present at high levels. Therefore, new cardioprotective approaches are crucial to improve the health of patients. To date, experimental investigations of acute ischemia-reperfusion injury (IRI) have generally demonstrated the efficacy of local ischemic preconditioning and postconditioning cardioprotection techniques as well as of remote conditioning. However, application in clinical settings is still highly controversial and debated. Currently, remote ischemic conditioning (RIC) seems to be the most promising method for heart repair. Protective factors are released into the bloodstream, and protection can be transferred within and across species. For a long time, the cross-function and cross-transmission mechanisms of cardioprotection were largely unknown. Recently, it has been shown that small, anuclear, bilayered lipid membrane particles, known as extracellular vesicles (EVs), are the drivers of signal transduction in cardiac IRI and RIC. EVs are related to the pathophysiological processes of cardiovascular diseases (CVDs), according to compelling evidence. In this review, we will first review the current state of knowledge on myocardial IRI and cardioprotective strategies explored over the past 37 years. Second, we will briefly discuss the role of EVs in CVD and the most recent improvements on EVs as prognostic biomarkers, diagnostic, and therapeutic agents. We will discuss how EVs can be used as a new drug delivery mechanism and how they can be employed in cardiac treatment, also from a perspective of overcoming the impasse that results from neglecting confounding factors.

## 1. Introduction

Worldwide, cardiovascular diseases (CVDs), ischemic heart conditions, and acute myocardial infarction (AMI) continue to be the primary causes of mortality and morbidity [1]. A major cause of illness and death relies on myocardial blood flow arrest involving the coronary tree and depends on thromboembolism or atherosclerosis. Myocardial infarction (MI)-related morbidity and mortality have been shown to correlate with the extent of myocardial damage. Indeed, infarct size increases with the duration of ischemia [2,3,4]. By decreasing the period of ischemia, treatments that immediately reperfuse the tissue, such as coronary stenting and balloon angioplasty, have considerably improved patient outcomes [5,6]. Despite these improvements, AMI continues to be the major worldwide cause of death [5,7]. Indeed, the best method for minimizing the extent of a myocardial infarct and improving the clinical outcomes after AMI is early and effective myocardial reperfusion with thrombolytic treatment or primary percutaneous coronary intervention (PCI). However, ischemia/reperfusion injury (IRI) can result from the restoration of blood flow to the ischemic myocardium [8]. Extracellular vesicles (EVs) have been shown to be involved in signal transduction in cardiac IRI and remote ischemic conditioning (RIC). Novel cardioprotective methods are needed to maintain cardiac cells in a healthy status and prevent the occurrence of heart failure. Cardiovascular research has therefore investigated methods to minimize IRI, resulting in the development of cardioprotective strategies also allowing a better knowledge of the molecular aspects of IRI. Investigating new cardioprotective strategies to provide a solid foundation for future translation into clinical practice has been the challenge of recent years. In this brief review, we consider the mechanisms of cardiomyocyte death triggered by IRI and then the complex signaling pathways elicited by ischemic conditioning, which include modification of sarcolemmal receptors and cytosolic kinases, as well as modulation of redox aspects, Ca^2+^ ions overload, mitochondrial permeability transition pore (mPTP) formation, and proteolysis. Finally, we will discuss the putative role of EVs in CVDs and cardioprotection and the most recent improvements on EVs as prognostic and diagnostic biomarkers. We will also discuss how EVs can be used as a novel drug delivery mechanism and how they can be used in cardiac treatment. We hope that a better understanding of EVs can help to overcome the impasse due to neglecting confounding factors, such as age, sex, comorbidities, and comedications (for reviews, see [9,10,11,12,13]).

## 2. Ischemia/Reperfusion Injury

The heart is responsible for the continuous supply of blood and nutrients to the entire body and to itself and does so through efficient pumping activity. This strenuous work involves the recycling of at least 6 kg of adenosine triphosphate (ATP) per day in a resting subject. Most ATP is produced through the aerobic oxidation of fatty acids [14]. Hence, the myocardium has a high oxygen demand, which is closely related to organ performance, and, therefore, disruption of oxygen supply has acute functional effects on cardiac activity: the myocardium does not tolerate hypoxia. Hence, heart cells die rapidly in cases of deficient blood supply through several mechanisms of death, but cells also die when blood flow is restored, and that is reperfusion injury [15,16]. The mechanisms that lead to the death of these cells are diverse. Mitochondrial-dependent and -independent pathways of cell death during cardiac IRI have been recapitulated by Davidson et al. [17]. In this brief review, we will consider some of them.

### 2.1. Pathophysiology of Myocardial Ischemia/Reperfusion Injury

Myocardial hypoxia can result from the reduction in coronary blood flow, which causes several metabolic and functional alterations, including mitochondrial electron transport chain inefficiency. Reduced ATP synthesis in mitochondria causes anaerobic metabolism, defective sodium–potassium pumps, and ribosome detachment from the endoplasmic reticulum [18,19]. ATP and antioxidants are produced at reduced rates during anaerobic metabolism in the cell. Therefore, during hypoxia, the myocardium forms and consumes less ATP. Hence, retention of sodium in cells and potassium outflow are caused by defective Na^+^-K^+^-ATPase pumps. Since the quantity of sodium in the cell increases and its transmembrane gradient is reduced, the sodium–hydrogen (Na^+^-H^+^) exchanger (NHE) and sodium–calcium (3-Na^+^/1-Ca^2+^) exchanger (NCX) become less active and intracellular Ca^2+^ and H^+^ increase. Moreover, because of ATP deficiency, malfunction on the cell surface of Ca^2+^-ATPase pumps and on the endoplasmic reticulum of Sarco-Endoplasmic Reticulum Calcium ATPase 2 (SERCA2), Ca^2+^-ATPase pumps promote *calcium overload*. Hydrogen, sodium, and calcium ion excess in cells results in hyperosmolarity, which promotes water flow into the cytoplasm and cell swelling. The retention of hydrogen ions and lactic acid might result in metabolic acidosis. Hence, low intracellular pH (pHi) impairs enzyme function and causes nuclear chromatin to clump next to each other. On the other hand, calcium overload favors calpain activation, hypercontracture (contraction band necrosis) and mPTP opening/formation. In addition to the switch to anaerobic metabolism and the dysfunction of ionic pumps, reduced ATP production contributes to the aforementioned ribosome detachment, negatively affecting protein synthesis [18,19]. Later, during the reperfusion phase, blood flow replenishes the ischemic tissue with nutrients and oxygen. Since cells in the ischemic phase have reduced concentrations of antioxidant agents, they undergo increased production of *reactive oxygen species* (ROS) when oxygen returns [15]. ROS generate oxidative stress, driving DNA damage, endothelial dysfunction, and local inflammatory reactions. *Calcium overload* exacerbates mitochondrial dysfunction, and cell death also driven by cellular structural damage may occur through several mechanisms [17].

#### 2.1.1. Oxidative Stress

It is commonly assumed that ROS are the main culprits behind macromolecular deterioration and represent the harmful byproducts of aerobic metabolism. Both ischemia and reperfusion can favor a “burst” of ROS production from the mitochondria [20,21]. Although early reperfusion is the most effective strategy to save ischemic tissue, as mentioned above, restoration of blood flow can promote pathological processes that cause greater myocardial damage than the primary ischemic insult [8]. Reperfusion is associated with an increase in ROS production [22], but it is unclear where these ROS originate or if they play a critical role in myocardial damage [23,24]. In isolated cardiomyocytes, sub-lethal hydrogen peroxide generation during simulated ischemia contributes to cell death during reperfusion, which is mediated by the reperfusion oxidant “burst” [25]. It has been demonstrated that oxidative stress during ischemia primes cardiomyocytes towards cell death during reperfusion. Nevertheless, ROS and increased calcium are thought to work together to cause the opening of mPTP, which is considered a key mediator of reperfusion injury [26]. Of note, pathological conditions involving the onset of oxidative stress, such as diabetes, make the heart more susceptible to ischemic damage and less likely to be protected by conditioning maneuvers [27]. An interesting finding for the scope of this review is that diabetes impairs RIC-induced cardioprotection by exosomes [28,29].

In summary, myocardial IRI pathophysiology is characterized by oxidative stress because ROS synthesis increases when oxygen supply is restored after ischemia. Nevertheless, while huge amounts of ROS are harmful, especially in the presence of comorbidities, and can cause or contribute to cardiomyocyte death, low levels of oxidants can be cardioprotective in conditioning approaches [23,24].

#### 2.1.2. Calcium Overload

Adequate intra-sarcolemmal Ca^2+^ levels in myocardial fibers, in addition to contractile function, are essential for cell survival. A series of primary and secondary active mechanisms operate to extrude free Ca^2+^ from the cytoplasm and transport it outside the cells. Transporters and pumps located on the plasma membrane, as well as the sarcoplasmic reticulum and SERCA2 pump, which transports Ca^2+^ within the reticulum itself, and mitochondrial Ca^2+^ transporters play important roles in keeping calcium levels under tight regulation within cardiomyocytes and organelles [30].

Ischemia and hypoxia disrupt the natural equilibrium of cells during the ischemia–reperfusion damaging process, which leads to an inadequate cell energy supply. As stated above, the energy-dependent Na^+^-K^+^-ATPase are less active when ATP levels are low, so intracellular levels of Na^+^ increase and NCX slows down. Therefore, increased intracellular Ca^2+^ concentration is the consequence of compensatory ion exchange; precisely, Ca^2+^ overload during ischemia is mainly due to the slowing down of the NCX, whereas during reperfusion it is largely due to the entry of this ion through the same exchanger. Indeed, NCX can also work in reverse mode as early as during the preceding ischemia, but especially during the first few minutes of reperfusion. The reverse operation of NCX is the consequence of an altered trans-membrane Na^+^ gradient, which results also from an increase in intracellular Na^+^ concentration associated with pHi correction mechanisms by NHE. An elevation in glycolytic processes accompanying lactic acid production, acidification of the cytosol, and a drop in pHi is seen after a hypoxic/ischemic event because cells remain in ATP deficiency and cannot oxidize fatty acids due to mitochondrial dysfunction. Due to a constant accumulation of Na^+^ within the cell, NHE does not effectively balance the increase in hydrogenation during ischemia. A functioning deficit of the ATPase pumps promotes additional Na^+^ buildup. This accumulation, as said, causes the NCX to change direction, especially during reperfusion, thus increasing the level of Ca^2+^ inside the cells which is made worse by malfunctioning of the Ca^2+^-ATPases, as well as by reperfusion, which washing out extracellular H^+^ speeds up the NHE, further increasing Na^+^ entry and altering the trans-membrane Na^+^ gradient.

Increased ROS, the destruction of cell membranes, and interference with mitochondrial function are just a few of the mechanisms involved in calcium excess-mediated cell damage [30], which also leads to enzyme activation (e.g., calpain). These alterations last until ATP synthase, Na+/K+-ATPase activity, and membrane potential are restored, otherwise cell death inflammatory processes occur.

#### 2.1.3. Nitric Oxide and Nitroxyl

Other key players in the ischemia/reperfusion scenario are nitric oxide (NO) and its sibling nitroxyl (HNO). NO is a well-known messenger involved in the relaxation and vasodilation of vascular smooth muscle and can induce protein modification through several metabolic pathways. The half-life of NO is only a few seconds, making it incredibly sensitive and labile. It has been asserted that it can prevent mPTP opening at physiological concentrations [31]. Numerous studies indicate that the NO-cyclic guanosine monophosphate (cGMP) pathway has a significant protective function against IRI [32]. cGMP is produced when NO binds to soluble cGMP. Then, cGMP stimulates protein kinase G (PKG), which controls several of NO’s activities, including mitochondrial function and mPTP opening. When pathological conditions are present, NO production, either insufficient or excessive, results in detrimental events. HNO donors also inhibit mPTP opening, thus limiting myocyte loss upon reperfusion, a beneficial effect that requires translocation of protein kinase C epsilon isoform (PKCε) into mitochondria whereas activation of mitochondrial K^+^ channels may be there with NO but not with HNO [33].

#### 2.1.4. Mitochondrial Permeability Transition Pore Opening

The revision of current knowledge stems from the discovery that mitochondria are not only the main energy producers of the cell, but also play a crucial role in the decision to live or die [34]. It is well-established that mitochondrial dysfunction is a key factor in the pathophysiology of numerous cardiac disorders, including IRI. Although the exact molecular nature of mPTP is unknown, it is recognized that extensive and long-duration mPTP opening mediates the fatal permeability alterations that initiate mitochondrial-dependent death in these disorders [35].

##### Events Triggering the mPTP Opening

Numerous pathophysiological events boost the mPTP opening. High mitochondrial Ca^2+^ concentrations ([Ca^2+^]m), in particular, can produce mitochondrial calcium overload-related defects that are easily noticeable in cardiac cells, including mitochondrial membrane potential (ΔΨm) depolarization and reduction of ATP production [36]. Extra [Ca^2+^]m, together with ROS, contributes to the binding of cyclophilin D (CyPD) to the inner mitochondrial membrane (IMM), promoting the formation of mPTP and possibly sustains its role in determining the heart damage [37]. Additionally, elevated [Ca^2+^]m results in an excessive increase in ROS production, which oxidizes IMM lipids and proteins. The mPTP opening ensures that this oxidation enhances the non-selective IMM permeability, which causes the IMM to enlarge and lose the cristae surface before irreversible mitochondrial malfunction results in cell death. Opening of mPTPs is caused by reperfusion of the heart following ischemia.

##### Consequences of the mPTP Opening

Firstly, the IMM is impermeable to all but a few specific compounds and ions under normal circumstances. However, under stressful circumstances, the mitochondrial inner membrane’s mPTP opening can occur, allowing any molecule smaller than 1.5 kDa to freely enter [38]. All small molecular weight solutes penetrate the membrane readily when the mPTP opens, but proteins do not, and as a result, they exert a colloidal osmotic pressure that causes mitochondria swelling [39]. Secondly, protons can pass easily through the inner membrane. As a result, oxidative phosphorylation becomes decoupled [40]. The proton-translocating ATPase then actively hydrolyzes ATP instead of synthesizing it. This causes the disruption of ionic and metabolic balance and the activation of degradative enzymes such as phospholipases, nucleases, and proteases. Intracellular ATP concentrations also drop quickly under such conditions. Without pore closure, these modifications will irreversibly harm the cell, leading to necrotic death. Even if closure does take place, the swelling and outer membrane breach in the mitochondria may be enough to start the apoptotic cascade.

##### Transient and Long-Lasting mPTP Opening

The mPTP is maintained tightly closed under normal physiological circumstances while activated under pathological conditions. Actually, it has been proposed that transient mPTP opening occurs without affecting cell viability [41], and that even these transient mPTP openings may contribute to the cardioprotection induced by ischemic preconditioning (see below) [42]. However, there are currently no specific techniques or interventions to distinguish between short- and long-lasting openings, especially in entire hearts (ex vivo or in vivo).

As mentioned above, the principal reason for mPTP’s long-lasting opening is mitochondrial calcium overload, which occurs when the amount of calcium in the mitochondrial matrix is significantly increased. This is especially true when other factors such as oxidative stress, adenine nucleotide depletion, high phosphate concentrations, and mitochondrial depolarization are present [38,40]. There is growing evidence that mPTP’s long-lasting opening is crucial in the change from reversible to irreversible reperfusion damage since these circumstances are precisely what the heart goes through during post-ischemic reperfusion [38,40].

ATP synthase has recently been shown to function as the primary “uniporter” of mitochondrial K^+^ ions, i.e., the main pathway of K^+^ entry into mitochondria. Thus, both H^+^ and K^+^ ions entry is proportional to ATP synthesis, regulating matrix volume and the matching of energy supply and demand. This discovery could be of revolutionary importance in the context of cardioprotection [43,44].

## 3. Cardioprotective Strategies

Numerous therapies that give strong cardioprotection in experimental animal models of acute ischemia and reperfusion damage have been identified. However, there has been a distressing lack of success in implementing these cardioprotective medications for patient benefit in the clinical setting of AMI. On this topic there are several reviews; the reader can start from the elegant recent reviews by COST Action EU-CARDIOPROTECTION CA16225 [10,13]. The fact that AMI is multifactorial and affects various cell types, including immune cells, platelets, fibroblasts, endothelium, and smooth muscle cells, in addition to cardiomyocytes, may play a significant role. In individuals with comorbidities and/or co-medications, cardioprotective strategies may not be protective because some protective pathways may be impaired; yet, many cardioprotective strategies considering combinations of medications may not be as effective since they use similar targets and end-effectors. Nevertheless, recent research indicates that combining additive or synergistic multitarget therapy (targeting multi-cells, -pathways, -strategies) may be necessary for the best cardioprotection [11]. Numerous cardioprotective methods to prevent myocardial IRI have been offered during the past three decades [45]. Based on the protective mode, time of administration, and cellular and intracellular target, they may be generally categorized into several groups. The cardioprotective techniques that have undergone the greatest research involve either the application of brief, carefully controlled bouts of ischemia and reperfusion (ischemic conditioning), the administration of chemicals (pharmacology), or the use of physical interventions, including thermic approaches. Ischemic conditioning, including local pre-conditioning (IPC), post-conditioning (IPostC), and RIC, is the backbone of cardioprotective therapies against ischemia reperfusion damage in preclinical studies. Although the mechanisms of ischemic conditioning are only partially known, they are likely to be numerous. Cardioprotective approaches can also be described based on the moment they are used, i.e., prior to, during, or following ischemia. Prior (IPC), during (per-conditioning, PerC), or right after (IpostC) an ischemic event are the possible applications of the conditioning stimuli.

### 3.1. Target for Cardioprotective Therapies

By their ultimate goals/targets, cardioprotective therapies can be further divided: ion exchangers and channels, proteases, ROS, contractile elements, or components of the mPTP are some examples of molecular targets under the first category that are primarily implicated in necrotic cell death. These approaches have hardly ever advanced to clinical trials and have often focused on the use of currently available pharmacological agents [10,11,13]. Cyclosporine A, specifically targeting the mPTP, represents a peculiar case. Cyclosporine A, indeed, had conflicting preclinical outcomes and was ineffective in large clinical studies. Acute myocardial IRI may also trigger other types of cell death, such as apoptosis, autophagy, necroptosis, and pyroptosis, all of which may influence the size of the subsequent MI to variable degrees and offer potential new targets for cardioprotection. The NO/cGMP/PKG cascade, Reperfusion Injury Salvage Kinase (RISK), and Survivor Activating Factor Enhancement (SAFE) pathways, mitochondrial morphology, and cardiomyocyte metabolism are examples of targets in the second category. Last but not least, cardioprotective techniques can either rescue cardiomyocytes or non-cardiomyocyte cells including platelets or leukocytes [46]. The myocardium comprises a variety of different cell types, particularly endothelial cells, fibroblasts, smooth muscle cells, and neuronal cells, which seem to be relevant actors in myocardial IRI, despite cardiomyocytes being the working cells in the heart and the most vulnerable to IRI. Of note, microRNA (miRNA) and exosomes, secreted mostly by endothelium and fibroblasts (i.e., the “secretome”) may support cardioprotective signaling [47,48,49,50,51].

### 3.2. Pre-Conditioning

Beginning in 1972, Braunwald and coworkers reported that the infarct size may be decreased by cardioprotective measures in addition to being dependent on the length of ischemia, the area at risk, and collateral flow [52]. Only fourteen years later, in 1986, Murry et al. [53] observed in an animal model that a brief period of coronary occlusion followed by a brief period of reperfusion decreased the area of MI brought on by a later protracted fatal ischemia/reperfusion phase [33]. A preconditioned state was produced via protective signal transduction, as demonstrated by Liu et al. in 1991 [54]. In fact, it has been demonstrated that several cardiac Gi-coupled receptors may stimulate IPC [55]. The heart develops a protective state that is identical to IPC when adenosine or an adenosine A1 receptor-selective agonist is injected into the coronary arteries for five minutes before occluding a coronary branch. On the other hand, a non-IPC heart was unaffected by an adenosine receptor antagonist, which prevented IPC protection. In contrast with adenosine A2 receptors, which are Gs-coupled and work to dilate the coronary arteries, A1 receptors are Gi-coupled and function to reduce the heart rate. Actually, the Consortium for preclinicAl assESsment of cARdioprotective medicines (CAESAR), a multicenter network of experimental research centers, has shown the infarct size-limiting effects of IPC in all examined species, including mouse, rat, rabbit, cat, dog, sheep, baboon, and humans, and has also proved that these treatments are beneficial [56,57].

In general, infarct size, apoptosis, endothelial dysfunction, and activation, as well as neutrophil adhesion and inflammation are all generally decreased by IPC. It also lessens arrhythmias and stunning [58]. Additionally, IPC decreases mitochondrial metabolism while promoting endothelial function and vascular response to endothelium-dependent vasodilatation [58,59,60,61,62]. IPC was initially described as an instantaneous adaptation of the heart to relatively short coronary occlusions [63]. Subsequently, IPC has been found to be a biphasic phenomenon, with an initial protective phase that develops minutes after the initial ischemic insult and lasts 2 to 3 h, and a late (or delayed) phase that appears 12 to 24 h later and lasts 3 to 4 days. The electrocardiogram (ECG), infarct size, cardiac contractility, frequency of arrhythmias, and biochemical tests, such as troponin or creatine kinase release, are some of the methods used to evaluate the effectiveness of IPC stimulation. Nevertheless, the evaluation of the reduction of the infarct size is the standard method to estimate myocardial healing in preclinical studies. Adenosine, bradykinin, and opioids are among the numerous defensive chemicals generated by the myocardium after a brief preconditioning ischemia that work together to induce the preconditioned state by activating PKC. While bradykinin and opioids couple to PKC through a complicated route that comprises, in order, Akt, NO synthase, guanylyl cyclase, PKG, opening of mitochondrial ATP-sensitive potassium channels (KATP), and activation of PKC by redox signaling, adenosine couples directly to PKC through the phospholipases. Even the opioid and bradykinin coupling are different from one another; the former stimulates phosphatidylinositol 3-Kinase (PI3K) via transactivation of the epidermal growth factor receptor, whilst the latter has an unidentified coupling mechanism. Protection results from the activation of the survival kinases Protein Kinase B/Akt (PKB/Akt) and Extracellular Signal-Regulated Kinases (ERK), which prevent the early stages of reperfusion from leading to the formation of mPTPs. Since PKC in some way favors communication from adenosine A2 receptors early in reperfusion, these kinases must be active to obtain cardioprotection. By phosphorylating Glycogen Synthase Kinase-3β (GSK-3β), the survival kinases are considered to prevent the formation of mPTP. The protective pathways operate for only a couple of hours and then fade away, in the so-called first window of protection, only to reappear after 24–48 h in the so-called second window of protection [64].

### 3.3. Postconditioning

The major limitation of IPC for cardioprotective therapy is the need to act before the ischemic event occurs, which is impossible to predict in the case of AMI. By interrupting myocardial reperfusion with a few short episodes (a few seconds) of myocardial ischemia, Zhao et al. in 2003 obtained the remarkable finding that the heart might be protected against IRI. This process is known as “ischemic postconditioning” [65,66]. The researchers discovered that administering three cycles of 30 s left anterior descending (LAD) coronary artery occlusion and reflow within one minute of myocardial reperfusion reduced MI size in canine hearts by 44%. IPostC was found to provide a wide range of benefits in addition to limiting MI, including decreased myocardial edema, oxidative stress and polymorphonuclear neutrophil buildup, as well as the retaining of endothelial function. These results were in line with postconditioned hearts’ lower myocardial reperfusion damage [66]. In contrast with the IPC stimulus, which consists of one to four short ischemia–reperfusion cycles (e.g., four 5-min ischemias spaced five minutes apart), the IPostC stimulation is brief, lasting only three to six cycles of ischemia and reperfusion (most often of 5–60 s ischemia duration separated by 5–60 s reperfusion). For a change in mitochondrial function in IPC, short bouts of ischemia and reperfusion are necessary, but in IPostC, the intermittent or stuttering reperfusion is likely the most crucial component of the procedure. Small animal cardiac models of IRI require intermittent ischemia and reperfusion conditioning periods of 5–10 s, while larger animal models and humans require IPostC treatments lasting 30 to 60 s [67]. It has been suggested that postconditioning during coronary angioplasty protects the human heart in a multicenter, randomized, controlled study. The research included thirty individuals who had coronary angioplasty for an ongoing AMI. As a consequence of four one-minute occlusions of the affected coronary arteries, the IPostC group’s infarct area was reduced by 36%, according to the results, which were caused by the balloon used for the angioplasty. Since then, several other small studies in humans have confirmed protection from ischemic postconditioning. However, large randomized trials have failed to demonstrate any benefit, although they have not reported deleterious effects [10,13].

### 3.4. Remote Ischemic Conditioning

An intriguing phenomenon, known as RIC, is induced by short periods of ischemia and reperfusion applied to a vascular bed, tissue, or organ. These ischemia/reperfusions confer general protection and make tissues and organs distant from the point of application resistant to damage caused by prolonged ischemia followed by reperfusion. RIC, about three decades ago, was first observed in the heart [68]. These brief periods of distant ischemia/reperfusion can occur before (preconditioning), after (postconditioning) or simultaneously (preconditioning) with the prolonged coronary occlusion that causes the infarction [69]. RIC has been demonstrated to be effective primarily by the reduction of infarct size [47,65,70]. Of course, conditioning does not offer protection when the coronary arteries are permanently occluded; instead, it only works when there is subsequent reperfusion, limiting the so-called IRI [71]. Via humoral [72] and neuronal paths from the distant stimulus site, RIC activates various defensive mechanisms in the cardiovascular system that are comparable to those that are engaged by local preconditioning. Additionally, RIC reduces platelet activation, endothelial impairment, and the inflammatory process in response to IRI [73,74,75,76]. The neural process and timeline of remote ischemic preconditioning (RIPC) of human endothelium were examined by Loukogeorgakis et al. [77]. The study confirmed that RIPC also acts in two stages to protect endothelial cells against ischemia damage in arterial vessels: an initial phase that is engaged right away and lasts up to four hours, and an additional phase that begins after 24 h of RIPC stimulation and lasts for at least 48 h. The administration of the autonomic ganglion blocker trimethaphan (16 mg/min intravenous infusion), which decreased both the early and delayed stages of RIPC, demonstrated that both phases of protection were dependent on the preservation of autonomic function. It has been found that the formation of free ROS increases during remote preconditioning, which may cause redox signaling to provide cardioprotection [78,79,80,81]. In order to provide cardioprotection during the ischemic phase, the sudden ROS increase during RIPC may enhance the production of heat shock proteins (HSP-70, HSP-25) that provide cardioprotection by reducing oxidative stress. This brief spike in ROS also elevates antioxidant enzymes (Mn superoxide dismutase and glutathione peroxidase) inside the stressed heart areas [82]. Additionally, redox signaling may have an impact on genes that regulate inflammation, such as Early growth response-1 (Egr-1), and a reduction in Egr-1 gene expression may lessen ischemia damage and provide cardioprotection [83]. An intriguing systematic review and meta-analysis found that none of the experimental factors examined by meta-regression could account for the high variability in RIPC-induced cardioprotection [84]. The research showed that studies lack uniformity in terms of study design and quality. Therefore, there is a critical need for more thorough in vivo research, with an emphasis on thoroughly describing RIPC/RIC methods and examining the possible effects of comorbidities. The CONDI2/ERIC-PPCI trial, the most current and pertinent study that impacts negatively on the efficacy of the RIPC procedure, showed that patients who received the RIPC therapy lacked adequate myocardial protection [85]. The importance of the release of circulating cardioprotective triggers in response to RIPC has been confirmed in a recent study, which also highlights that the lack of response to circulating cardioprotective factors may be due to metabolic alterations within the myocardium [86]. 

It is clear that remote conditioning culminates in a systemic response. To this end, further investigation is needed to determine whether there are many more factors capable of transmitting the conditioning stimulus to properly apply the cardioprotection of remote conditioning to patients with CVDs. Even though humoral and neuronal responses have been attributed to the conditioned response of RIC, it is still unexplained how cardioprotective signals are shared between organs at this time. Particularly, it has recently been established that EVs contribute as sources of signal transduction in cardiac IRI and RIC [87]. Similar circumstances exist to EVs that are released from the heart after ischemia preconditioning [28]. In addition, Ma et al. also observed that following RIC procedures in rats, there is a quick rise in cardioprotective microparticles, primarily originating from platelets [88]. Goals for applied research have been greatly boosted by the finding that EVs take part in the transmission of information in physiological and pathological settings. Similar to RIC, drugs may modify EVs content. For instance, Silva-Palacios and colleagues investigated if citicoline alters the composition of exosomes in patients with AMI undergoing coronary angioplasty to see if these vesicles may be transmitters of cardioprotective agents. They discovered that this drug alters certain miRNAs that are involved in cardioprotection. In particular, cell death increased in the presence of exosomes from infarcted patients, whereas incubation of cardiomyoblasts with exosomes from patients treated with citicoline did not affect cell viability [89]. The need for more study is emphasized by these preliminary findings, especially to discover new tools that will allow researchers to generate novel ideas and conduct their work with greater accuracy and precision. However, until we have a clearer understanding of the molecular processes taking place at the cellular and subcellular levels, the EVs signaling field will not be able to stand on its own.

## 4. Extracellular Vesicles: Novel Communication Concept for Cardioprotective Maneuvers

Mammals’ development and homeostatic maintenance depend on cell–cell communication, which enables quick and effective reactions to changes or dangers in the milieu that affect host cells. In addition to the traditional mechanisms of cell-to-cell interaction and the release of soluble compounds such as cytokines, inflammatory mediators, metabolites, and hormones, EVs are another form of intercellular communication [90].

**Figure 1:**
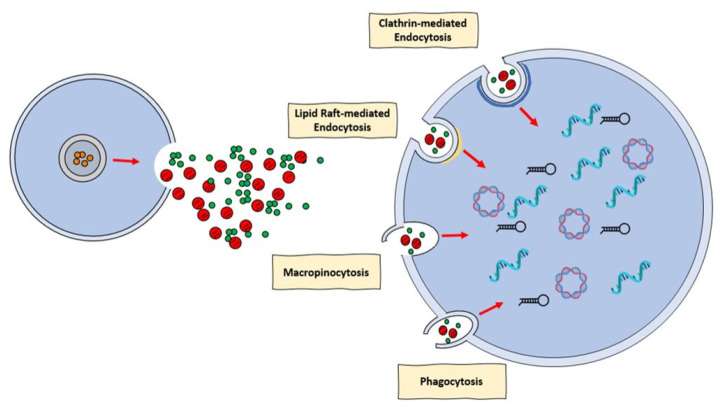
Mechanisms of release and uptake of extracellular vesicles (EVs). EVs that are released by practically all cell types, are categorized as small and medium-large. It has been demonstrated that clathrin or lipid raft-mediated endocytosis can take up EVs. As uptake processes, phagocytosis and macropinocytosis have also been noted. In the recipient cell, EVs release their cargo, such as miRNA, circRNA, and mRNA.

EVs are small, anuclear, bilayered lipid membrane particles that are released by practically all cell types. They are abundant in a variety of bioactive compounds, including lipids, proteins, amino acids, mRNAs, and miRNAs (Figure 1) [91,92]. Their composition essentially depends on the unique (patho)physiological condition at the time of EVs packing and secretion, in conjunction with the origin of the cells [91]. 

The term “extracellular vesicle” has been adopted by the International Society of Extracellular Vesicles (ISEV) as a standard name for particles that the cell naturally releases but cannot replicate and are confined by a lipid bilayer [93]. According to the ISEV, EVs can be categorized as small (less than 100 nm in diameter) and medium–large (greater than 100 nm in diameter). EVs can be released from several cardiovascular cell types including endothelial cells, cardiomyocytes, macrophages, and fibroblasts, but can also be released from extracardiac cells erythrocytes and platelets. Intriguingly, some EVs derived from the vasculature and some from cardiomyocytes may play a role in cardioprotection [94,95].

EVs may be recovered from a range of bodily fluids, including blood and plasma, and they have been demonstrated implicated in a number of pathophysiological and cardioprotection processes. As a result, it has been suggested that EVs can be used as both a disease biomarker and a curative agent [96,97]. To have this impact, EVs must integrate with recipient cell membranes, either physically with the cellular membranes or with the endosomal membrane following endocytic absorption. There is a huge amount of proof that EVs can penetrate cells and discharge their message. Nevertheless, there has been much disagreement in the literature regarding the mechanism behind the incorporation of EVs into cells [98]. Specifically, a number of processes, including clathrin-mediated endocytosis (CME), phagocytosis, macropinocytosis, and plasma or endosomal membrane fusion, have been described for EVs absorption (Figure 1). The many processes by which EVs can be assimilated by recipient cells were discussed in an intriguing review provided by Mulcahi and colleagues [99]. In that review, several methodologies were also highlighted using the uptake mechanism which was shown [99]. 

### Applications of EVs in Therapy and Their Roles as Prognostic and Diagnostic Biomarkers for CVDs

As also previously indicated, there is considerable evidence that EVs play a role in maintaining homeostasis; however, the focus now is on how EVs become dysregulated during illness [100].

**Figure 2:**
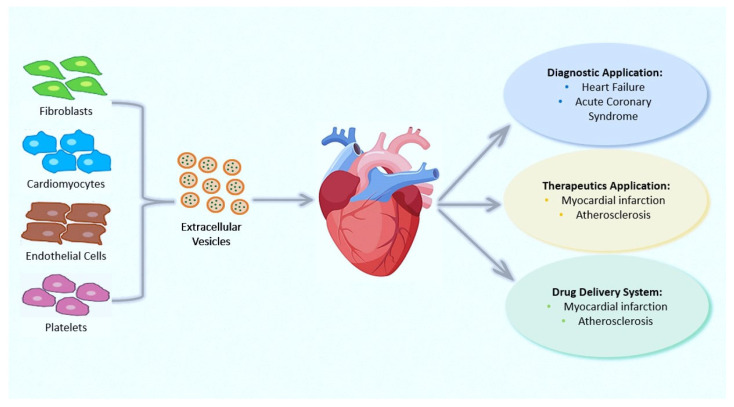
Implications of extracellular vesicles (EVs) in cardiovascular diseases (CVDs). Several cells release EVs that deliver their cargo to cardiac cells. EVs have been involved in diagnostics and therapeutics applications and drug delivery systems. EVs are a potential diagnostic and prognostic biomarker for CVDs, including heart failure, acute coronary syndrome, myocardial infarction, and atherosclerosis.

CVDs, which comprise a myriad of pathological conditions, including coronary artery disease (CAD), stroke, hypertension, and peripheral arterial disease, remain the leading cause of morbidity and mortality worldwide. EVs have a role in CVDs, communication involving cardiomyocytes, fibroblasts, smooth muscle cells, and endothelial cells, as well as the management of heart regeneration, ventricular remodeling, and angiogenesis (Figure 2). Numerous fundamental scientific and clinical investigations on EVs and cardioprotection have been conducted and the roles of EVs in CVDs have already been increasingly understood (for review see [101]). EVs can exert a plethora of effects in the ischemic and post-ischemic heart that lead to maladaptive remodeling, including fibrosis, angiogenesis, immune cell recruitment, and inflammation, but also to post-ischemic scar resolution [102,103]. EVs are also involved in atrial fibrillation (AF) progression. For instance, Shaihov-Teper et al. demonstrate a causal relationship between epicardial fat and AF, attributing a clear profibrotic, proinflammatory, and proarrhythmic effect of EVs derived from epicardial fat [104].

We recently summarized studies on the beneficial effects of EVs supplied by various cell types in the cardiovascular system in a review article published by our research team and other colleagues [96]. EVs govern pathological processes such as atherosclerosis, hypertension, and MI by releasing their content into the target cell as well as physiological processes such as tissue repair and coagulation [105,106]. Briefly, the most pertinent discoveries we have reported involve specific miRNAs delivered through EVs that have been considered as prognostic and diagnostic criteria in various clinical situations. In particular, recent studies have revealed a rise of circulating miRNAs in the plasma of subjects with AMI [107,108]. In contrast to the traditional markers, troponin, and creatine–kinase MB (CK-MB), miRNA-208a has been clearly found in all individuals suffering from AMI 4 h after the onset of chest pain [107]. Circular RNAs (circRNAs), which are widely carried by EVs, have gained attention more recently [108]. It has also demonstrated the existence of crosstalk among cardioprotective pathways, EVs, and conditioning maneuvers [109]. It has been proposed that EVs released from the heart after ischemia are necessary for cardioprotection induced by remote conditioning, highlighting the importance of vesicular transfer mechanisms and interaction within the heart in remote cardioprotection [109].

Some studies have proposed RNA in EVs as *biomarkers* of CAD (Figure 2); for example, recently, circRNA, circNPHP4 resulted to be upregulated in EVs derived from monocytes of CAD sufferers, being correlated with clinical–pathological status [110]. Transported proteins in EVs can also serve as biomarkers of CVDs. For example, plasma factor H-linked complement protein (FHR-1) was found to be elevated in subjects with atherosclerosis [111], while the EVs’ connexin-43 was found to be downregulated in patients with ST-elevation myocardial infarction (STEMI) [112].

As stated in Section 3.1, it is well-recognized that cardioprotection requires the activation of beneficial myocardium signals, which primarily activate the RISK and SAFE pathways [113]. For instance, the activation of several kinases, including PI3k-Akt, Mek/Erk [114,115,116], and the downstream effector, GSK3β [117], is required for the RISK pathway, which was initially characterized by Yellon and Hausenloy group [8,35,42]. Interestingly, the RISK is blunted by diabetes [118]. The Janus Kinase (Jak) and Signal Transducer and Activator of Transcription 3(STAT3) were discovered as a part of the SAFE cascade, which inhibits mPTP opening [119,120,121]. In addition, to act as a transcription factor, STAT3 also exerts pro-survival non-genomic effects resulting in the protection of the mitochondrial function from IRI. As part of the SAFE enhancing pathway, which converges on the mitochondria and affects their function by interacting with other cardioprotective pathways, STAT3 has emerged as a key player in cardioprotection. There is evidence to support STAT3 as a metabolic network promoter. The management of the electron transport chain, the formation of ROS, the homeostasis of Ca^2+^, and the suppression of opening the transition pore of mitochondrial permeability are just a few examples of processes that STAT3 may drive to preserve mitochondrial functions [122]. Pre-treatment of the rat heart with STAT3 antagonist led us to demonstrate that STAT3 is crucial for EVs-induced cardioprotection [49]. Interestingly, a recent study revealed that a hypoxia-inducible circRNA that is highly expressed in cardiac endothelial cells, namely circWhsc1, can be released from EVs and can activate tripartite motif 59 binding to STAT3, thereby increasing its phosphorylation and inducing cardiomyocyte protection and proliferation [123].

Bioengineering to improve the use of EVs as therapeutic tools is a rapidly expanding field and evidence for the possible therapeutic role of EVs in CVDs is growing [124]. Cardiovascular research is constantly focused on using EVs in a clinical context to treat CVDs or stop disease progression by delivering certain medications, proteins, miRNA, and functional genes. Promising findings have been found in attempts to discover cell-derived exosomes harboring cardioprotective compounds. Additionally, the development of EVs as a means of medicinal administration is being made possible by mounting evidence about the mechanisms used in EVs cargo sorting [125]. In this regard, modification of the cargo or surface area of EVs isolated or EVs enriched with miR-21 has been able to reduce apoptosis in the hearts of infarcted mice [126]. Cells overexpressing Chemokine receptor 4 (CXCR4) also release EVs enriched in this receptor exerting cardioprotective effects [127]. Conjugation of peptides to isolated EVs improved functional recovery of the heart [128]. In addition to the direct use of EVs as therapeutic agents, identifying the impact of currently used drugs on EV-mediated communication is also a fertile field [89].

Human health preservation has not yet benefited fully, although both pharmacological and mechanical techniques have consistently improved preclinical models [90]. Conversely to preclinical studies, the major challenges in applying cardioprotective medicines to humans come from the variety of endpoints, the complexity of estimating long-term effects, and the presence of co-morbidities in patients [129].

Other investigations in addition to this significant clinical trial, have recently uncovered several features on EVs and RIPC. A local inflammatory response can be “shaped” by EVs produced from an ischemic myocardium, and serum-derived EVs from patients that have received coronary artery bypass grafts have been reported to be enriched in miRNA-21 [130,131]. Furthermore, following RIPC in rats, Ma et al. [88] demonstrated a fast boost in cardio-protective micro-particles, primarily produced from platelets. In a recent study, Abel et al. [132] looked at EVs obtained from anesthetized subjects who had received RIPC and coronary artery bypass graft surgery. The results of this investigation showed that EVs generated in response to RIPC are beneficial towards H9c2 apoptosis caused by hypoxia. It was not explored how they affected hypoxia and reoxygenation (H/R) [131]. Moreover, besides the work by Haller et al. [133], which described the cell of origin of EVs in patients with STEMI and exposed to RIPC, no studies have been performed on the cardio-protective qualities of circulating EVs isolated from acute coronary syndrome (ACS) patients, regardless of whether they had undergone RIPC or not. Despite the most current research and efforts to exploit the potential cardioprotective capabilities of EVs, there is still much to understand. Firstly, it is crucial to examine how EVs react to conditioning stimuli, by means of their cargo. Once these mechanisms are known, it may be possible to prevent the delivery of dangerous molecules while favoring those that can activate protective mechanisms. We have recently shown in patients with ACS that both RIPC and PCI (involving periods of additional ischemia) and inflammatory conditions reprogram EVs load, changing the cardioprotective properties of EVs and thus affecting the functional capacity of RIPC [28,30].

## 5. Conclusions and Perspectives

The preconditioning studies that began 37 years ago have enabled us to better understand the mechanisms of IRI that we described above. As we have seen, myocardial IRI can result from an AMI produced by abrupt coronary obstruction. To reduce damage and myocardial scar development, resulting in unfavorable myocardial remodeling and heart failure, coronary perfusion must be restored. PCI and coronary artery bypass grafting have allowed early revascularization/reperfusion, thus minimizing the ischemic area of the myocardium. However, as is well known, reperfusion per se promotes intensified and accelerated myocardial damage, known as IRI. This process is largely amplified by inflammation through numerous mechanisms of interaction [96]. We have seen that conditioning strategies, including RIC, may or may not reduce infarct size depending on several characteristics, including sex, age, strain, and environmental aspects of the animals. Over the years, we realized that it was difficult to translate cardioprotective phenomena into the clinic, in part because we overlooked these confounding factors in our laboratory experiments, where we used healthy and generally male animals. Several recent reviews consider these confounding factors in cardioprotection (e.g., [9,10,11,12,13]).

Recently, Heusch’s group observed that RIPC does not reduce myocardial infarct size in a porcine model, but humoral factors are released from the protective strategy and induce—when transferred—a reduction in infarct size in isolated rat hearts [86]. The authors do not analyze the nature of transferable cardioprotective factors. However, this important study paves the way for future investigations that could better understand transferable factors, including EVs. EVs can potentially be markers of disease and therapeutic agents, and hopefully they can provide us with information on the role of various confounding factors.

Therefore, we can conclude with some recommendations: (1) in planning our future experimental studies on cardioprotection, we need to consider confounding factors, including age, gender, genetic background, and standard co-treatments; (2) in translating the results to the clinic, we must consider multi-target strategies and must study the right patient population and test the efficacy of treatments in high-risk patients [134]; (3) we need to identify reliable indicators of the effectiveness of our therapeutic interventions: can EVs be useful? Only well-designed future studies can answer this question.

## Data Availability

Not applicable.
